# Efficacy of Adding Immune Checkpoint Inhibitors to Chemotherapy Plus Bevacizumab in Metastatic Colorectal Cancer: A Meta-Analysis of Randomized Controlled Trials

**DOI:** 10.3390/cancers17152538

**Published:** 2025-07-31

**Authors:** Fumihiko Ando, Akihisa Matsuda, Yuji Miyamoto, Yu Sunakawa, Tomoko Asatsuma-Okumura, Yoshiko Iwai, Hiroshi Yoshida

**Affiliations:** 1 Department of Cell Biology, Institute for Advanced Medical Sciences, Nippon Medical School, Tokyo 113-8602, Japan; ando-f@nms.ac.jp (F.A.); t-asatsuma@nms.ac.jp (T.A.-O.); y-iwai@nms.ac.jp (Y.I.); 2 Department of Gastroenterological Surgery, Nippon Medical School, Tokyo 113-8603, Japan; hiroshiy@nms.ac.jp; 3 Department of Gastroenterological Surgery, Graduate School of Medical Sciences, Kumamoto University, Kumamoto 860-8556, Japan; miyamotoyuji@kumamoto-u.ac.jp; 4 Department of Clinical Oncology, St. Marianna University School of Medicine, Kawasaki 216-8511, Japan; y.sunakawa@marianna-u.ac.jp

**Keywords:** metastatic colorectal cancer, immune checkpoint inhibitor, bevacizumab, cytotoxic, chemotherapy

## Abstract

Background: Immune checkpoint inhibitors (ICIs) have limited efficacy in proficient mismatch repair (pMMR) and microsatellite stability (MSS) metastatic colorectal cancer (mCRC). Inhibition of vascular endothelial growth factor (VEGF) or cytotoxic chemotherapy can boost immunogenicity and has the potential to upregulate ICI efficacy. Methods: A comprehensive electronic literature search was conducted up to April 2025 to identify randomized controlled trials comparing cytotoxic chemotherapy plus bevacizumab with or without ICI. The primary outcome was progression-free survival (PFS), and secondary outcomes were overall survival (OS), objective response rate (ORR), and severe adverse events (AEs: grade 3 or more). A meta-analysis was performed using random-effects models to calculate hazard ratios (HRs) or odds ratios (ORs) with 95% confidence intervals (CIs). Results: Four studies involving 986 patients (With-ICI group, *n* = 651; Without-ICI group, *n* = 335) were included. The meta-analysis demonstrated a significant improvement in PFS in the With-ICI group compared with the Without-ICI group, with an HR of 0.82 (95% CI: 0.70–0.96, *p* = 0.01) without statistical heterogeneity. No significant improvements were observed between the With- and Without-ICI groups in OS and ORR meta-analyses, but the With-ICI group had a favorable trend in OS. A significant increase in serious AEs was not observed in the With-ICI group. Conclusions: This meta-analysis suggests a potential benefit of adding ICIs to chemotherapy plus bevacizumab in pMMR mCRC; however, the evidence remains preliminary and hypothesis-generating, warranting further investigation in biomarker-driven trials and clarification of long-term outcomes.

## Simple Summary

This systematic review and meta-analysis evaluated the efficacy and safety of adding immune checkpoint inhibitors (ICIs) to chemotherapy plus bevacizumab in patients with metastatic colorectal cancer (mCRC). While ICIs are known to be effective in mismatch repair-deficient (dMMR) tumors, their benefit in mismatch repair-proficient (pMMR) or microsatellite-stable (MSS) tumors remains unclear. We analyzed four randomized controlled trials comprising a total of 986 patients. The pooled analysis showed that the addition of ICIs significantly improved progression-free survival (hazard ratio 0.82; 95% confidence intervals (CIs): 0.70–0.96; *p* = 0.01), with no significant increase in severe adverse events. However, no significant improvement was observed in overall survival or objective response rate. These findings suggest that ICIs may offer modest clinical benefit in combination with chemotherapy and bevacizumab in mCRC, including pMMR cases. Further studies are warranted to validate these findings and to identify biomarkers for selecting patients most likely to benefit from this approach.

## 1. Introduction

In recent years, immune checkpoint inhibitors (ICIs) have been incorporated into treatment strategies for metastatic colorectal cancer (mCRC), specifically for the subset of patients—approximately 5%—whose tumors exhibit deficient DNA mismatch repair (dMMR) and high microsatellite instability (MSI-H) [1]. The KEYNOTE-177 trial was a phase 3 randomized controlled trial (RCT) study comparing pembrolizumab as a monotherapy with standard chemotherapy in patients with MSI-H/dMMR metastatic colorectal cancer [2]. Pembrolizumab significantly improved progression-free survival (PFS) and showed a favorable safety profile, establishing it as an effective first-line treatment option for this patient population [2].

Approximately 95% of patients with mCRC exhibit proficient mismatch repair (pMMR) and microsatellite stability (MSS) [3]. Monotherapy with programmed cell death-ligand 1 (PD-L1) inhibitors has shown minimal efficacy in MSS mCRC [3]. Thus, there remains a significant unmet need for therapeutic combinations that can boost the clinical efficacy of anti-PD-1/PD-L1 antibodies in mCRC, especially for MSS. Dual inhibition of the vascular endothelial growth factor (VEGF) and PD-1/PD-L1 axes has resulted in therapeutic activity in multiple tumor types [4,5,6]. Nevertheless, combining PD-L1 inhibitors such as atezolizumab with VEGF inhibitors such as bevacizumab may counteract VEGF-driven immunosuppression, facilitate dendritic cell maturation, and enhance T-cell infiltration into tumors, as demonstrated by existing preclinical studies [7,8]. Furthermore, ICIs have been combined with cytotoxic chemotherapy to take advantage of the method’s tumor-killing capacity and to promote the release of neoantigens, which can initiate T-cell priming. On the basis of these preclinical studies with therapeutic potential, several clinical trials were conducted in mCRC [9,10,11,12,13]. AtezoTRIBE, a phase 2 RCT of 218 patients with mCRC in the first-line setting, which compared FOLFOXIRI plus bevacizumab with or without atezolizumab, demonstrated a significant improvement in PFS (median: 13.1 months vs. 11.5 months) [9,10]. However, the other studies found no significant results, and clinical questions remain open [11,12,13].

Although these trials have provided important insights, the clinical efficacy of combining ICIs with chemotherapy and bevacizumab in mCRC remains inconclusive, especially in pMMR tumors. Individual studies lacked sufficient power to confirm survival benefits, and heterogeneity in patient populations and regimens has further complicated interpretation.

We conducted a systematic review and meta-analysis to draw definitive conclusions around the additional efficacy of ICI administration with chemotherapy plus bevacizumab in mCRC patients with sufficient samples and with statistical power at this optimal timing.

## 2. Materials and Methods

This systematic review and meta-analysis were performed in compliance with the PRISMA 2020 guidelines for reporting systematic reviews and meta-analyses [14]. Institutional ethical review was waived because of the nature of the study design [15]. This systematic review and meta-analysis were registered with UMIN-CTR (ID: UMIN000057763).

### 2.1. Literature Search

We systematically searched the literature using MEDLINE (via PubMed), the Cochrane Central Register of Controlled Trials (CENTRAL), Google Scholar, and ICHUSHI-Web, a Japanese database maintained by the Japan Medical Abstracts Society, from the inception of each database to April 2025; we did not include unpublished studies, conference abstracts, or grey literature in this analysis, following our predefined protocol. Articles written in English and Japanese were considered eligible. Only randomized controlled trials (RCTs) were candidates for analysis. The selected MeSH search terms were (“colon cancer” OR “colorectal cancer” OR “rectum cancer” OR “rectal cancer”) AND (“immunotherapy” OR “immune checkpoint inhibitor”) AND (“vascular endothelial growth factor” OR “bevacizumab”). Two authors (F.A. and A.M.) independently assessed the relevance of all retrieved studies. To ensure completeness, the reference lists of all relevant articles were manually screened for potentially eligible studies not captured by the initial database search. This process was iteratively performed until no new relevant records emerged.

### 2.2. Bias Risk Assessment

To evaluate the internal validity of the included RCTs, the Cochrane Risk of Bias (RoB) tool was employed. The Cochrane RoB tool is a standardized instrument developed to assess the risk of bias in RCT. It evaluates several domains, including random sequence generation, allocation concealment, blinding, incomplete outcome data, selective reporting, and other potential sources of bias. The grading of recommendations assessment, development, and evaluation (GRADE) methodology was applied for assessing the quality of evidence, and it was reported in the results [16].

### 2.3. Data Extraction Process

The inclusion and exclusion criteria were defined *a priori*. The inclusion criteria were as follows: (i) diagnosed as metastatic and/or unresectable CRC; and (ii) RCT comparing cytotoxic chemotherapy plus bevacizumab with or without ICI. The primary outcome was progression-free survival (PFS), and secondary outcomes were overall survival (OS), objective response rate (ORR), and serious adverse events (AEs) (grade 3 or more). Studies investigating pediatric patients and animal studies, as well as those not evaluating predefined outcomes, were excluded. Each extracted study was evaluated by two independent investigators (F.A. and A.M.) for inclusion and exclusion. The following data were extracted from the included studies: author, year, country, study design, institution, study duration, and number and characteristics of patients.

### 2.4. Statistical Analysis

Survival outcomes were summarized as the logarithm of hazard ratios (HRs) with 95% confidence intervals (CIs) using the generic inverse variance method [17]. HRs and 95% CIs were obtained directly from individual articles; if not reported directly, they were calculated from indirect data. The DerSimonian–Laird model was employed for dichotomous variables to compute pooled odds ratios (ORs) with 95% CI [18]. Because of the heterogeneity among the studies, a random-effects meta-analysis was conducted, producing a more conservative HR or OR estimate compared with the fixed-effects model. An HR of less than 1 was interpreted as favoring the experimental group for survival outcomes. For ORR, an OR greater than 1 indicated a favorable outcome in the experimental group; however, for AEs, an OR of less than 1 was considered favorable for the experimental group. The result was considered statistically significant if the 95% CI excluded 1. The meta-analysis was performed using Review Manager Version 5.4.1 (Cochrane Collaboration, Copenhagen, Denmark). Heterogeneity between the included studies was assessed using χ^2^ and I^2^ tests, with χ^2^ *p* < 0.05 and I^2^ ≥ 50%, respectively, indicating heterogeneity [19]. Publication bias was evaluated by visual examination of a funnel plot, with asymmetry formally assessed using Egger’s linear regression test [20].

## 3. Results

### 3.1. Literature Identification and Inclusion

**Figure 1:**
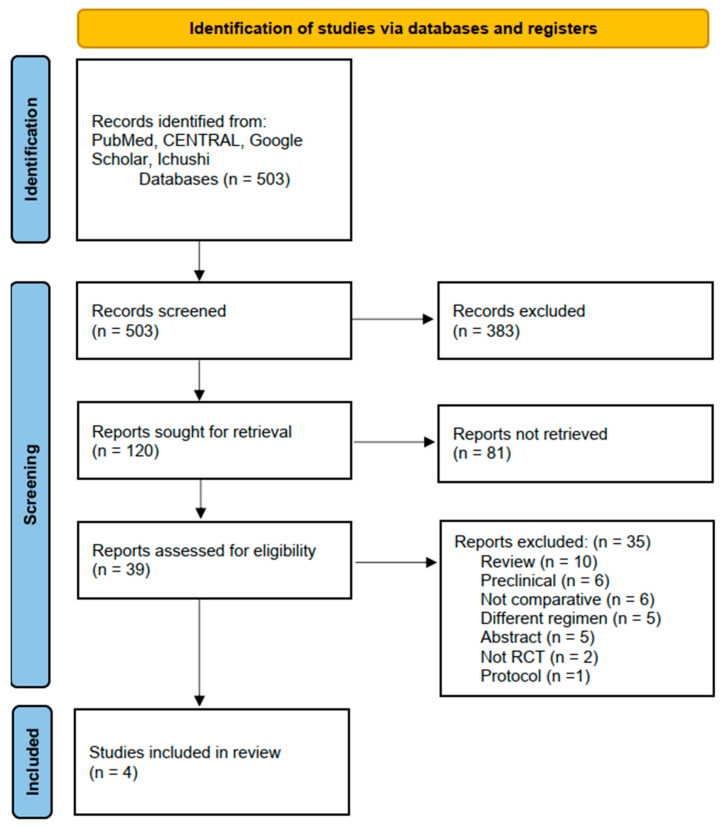
Flow diagram of included studies.

An initial literature search identified 503 records. After screening titles and abstracts, 39 articles were selected for full-text assessment, of which 35 were subsequently excluded following detailed review. Finally, the remaining four [9,10,11,12,13] studies (published between 2022 and 2024) were included in the meta-analysis. Figure 1 shows the PRISMA flow diagram summarizing the literature search and study-selection process. The background characteristics of the included studies are listed in Table 1. All [9,10,11,12,13] studies were published in English. Three [9,10,12,13] studies were phase 2 RCTs, and one [11] was a phase 2/3 RCT. Three [9,10,11,13] studies were open-label, while one [12] was double-blind. Three [9,10,12,13] studies adopted atezolizumab and one [11] nivolumab as ICIs. Three [9,10,11,13] studies were in first-line settings, and one [12] was in a second-line or later setting. The number of patients included in each study ranged from 113 to 445. Of the 986 patients included in the meta-analysis, 651 (66.0%) had cytotoxic chemotherapy plus bevacizumab with an ICI (With-ICI group) and 335 (34.0%) without an ICI (Without-ICI group).

### 3.2. Risk of Bias Assessment

A summary of the risk-of-bias (RoB) assessment using the Cochrane tool for the four included [9,10,11,12,13] studies is shown in Table 2. Three [9,10,11,13] studies were judged as having “some concerns” due to a lack of detailed information regarding blinding of participants and personnel, and one [12] was “low” in overall risk because it was only double-blinded. According to the GRADE criteria, the overall quality of evidence was “low” for all outcomes, including PFS, OS, and ORR (Table 3).

### 3.3. Primary Outcomes

**Figure 2:**
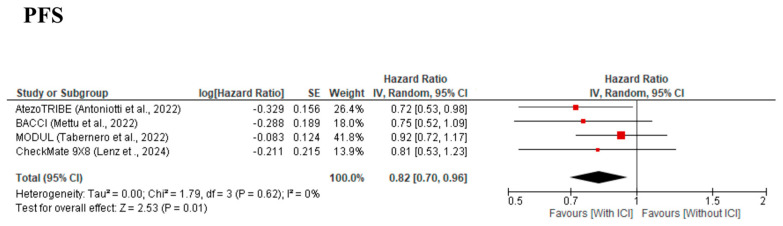
Forest plot comparing progression-free survival for cytotoxic chemotherapy plus bevacizumab with/without immune checkpoint inhibitors from four included studies [9,11,12,13].

PFS was evaluated in all four [9,10,11,12,13] studies. The meta-analysis demonstrated a significant improvement in PFS in the With-ICI group compared with the Without-ICI group, with an HR of 0.82 (95% CI: 0.70–0.96, *p* = 0.01) (Figure 2), and no significant between-study heterogeneity was observed (χ^2^ = 1.79, I^2^ = 0%, *p* = 0.62). The MODUL [13] and AtezoTRIBE [9,10] studies contributed the largest weights (41.8% and 26.4%, respectively), reflecting their relatively larger sample sizes and event counts. All individual HRs favored the With-ICI group, with all four studies showing HRs of <1.0, reinforcing consistency across trials. The narrow 95% CI of the pooled effect size further supports the statistical robustness of the observed benefit. Furthermore, no significant publication bias was detected by visual inspection of the funnel plot (Supplementary Figure S1A) or Egger’s test (*p* = 0.833).

### 3.4. Secondary Outcomes

**Figure 3:**
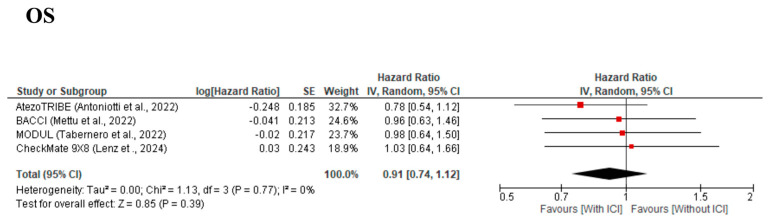
Forest plot comparing overall survival for cytotoxic chemotherapy plus bevacizumab with/without immune checkpoint inhibitors from four included studies [9,11,12,13].

**Figure 4:**
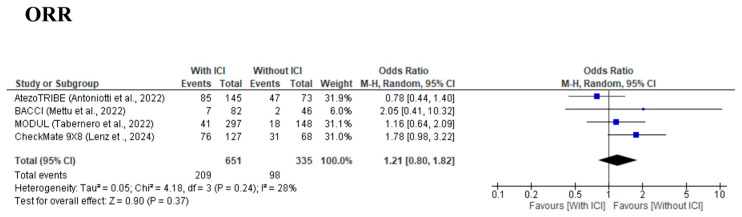
Forest plot comparing overall response rate for cytotoxic chemotherapy plus bevacizumab with/without immune checkpoint inhibitors from four included studies [9,11,12,13].

The meta-analysis for OS, which was assessed in four [9,10,11,12,13] studies, demonstrated a trend of better OS in the With-ICI group than in the Without-ICI group, but the difference did not reach statistical significance, with an HR of 0.91 (95% CI: 0.74–1.12, *p* = 0.39) (Figure 3), and no significant between-study heterogeneity was observed (χ^2^ = 1.13, I^2^ = 0%, *p* = 0.77). The forest plot revealed variability in study-level HRs: three studies (AtezoTRIBE [9,10], BACCI [12], and MODUL [13]) showed HRs of <1.0, while the CheckMate 9X8 [11] study had an HR of over 1.0. However, the CheckMate 9X8 [11] study had a wide confidence interval and minimal weight (18.9%). A significant publication bias was detected by visual inspection of the funnel plot (Supplementary Figure S1B) and Egger’s test (*p* < 0.001). The meta-analysis for ORR, which was assessed in four [9,10,11,12,13] studies, demonstrated no significant improvement, with an OR of 1.21 (95% CI: 0.80–1.82, *p* = 0.37) (Figure 4), and no significant between-study heterogeneity was observed (χ^2^ = 4.18, I^2^ = 28%, *p* = 0.24). No significant publication bias was detected by visual inspection of the funnel plot (Supplementary Figure S1C) or Egger’s test (*p* = 0.648).

### 3.5. Subgroup Analysis

Subgroup analyses were conducted to further explore potential sources of heterogeneity in PFS, specifically regarding treatment line settings and the class of immune checkpoint inhibitors (anti–PD-1 vs. anti–PD-L1 antibodies). In the first-line setting, which included three studies, the With-ICI group showed a significant improvement in PFS (HR = 0.83, 95% CI: 0.70–0.99, *p* = 0.04), whereas no significant benefit was observed in the subsequent-line setting, which included only one study (Supplementary Figure S2). Additionally, a subgroup analysis focusing exclusively on studies using anti–PD-L1 antibodies demonstrated a consistent improvement in PFS (HR = 0.82, 95% CI: 0.69–0.97, *p* = 0.02) (Supplementary Figure S3). However, no statistically significant subgroup differences were detected in either analysis, suggesting a similar therapeutic trend in the With-ICI group regardless of treatment line or ICI class.

### 3.6. Safety Profile

**Figure 5:**
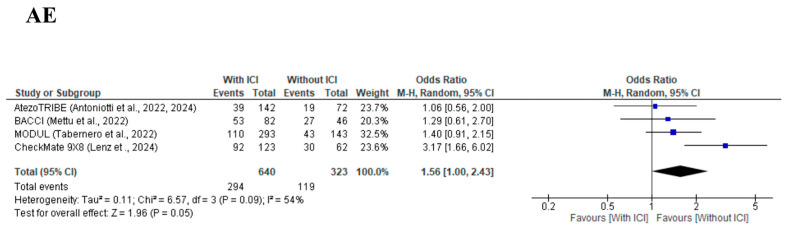
Forest plot comparing serious adverse events for cytotoxic chemotherapy plus bevacizumab with/without immune checkpoint inhibitors from four included studies [9,10,11,12,13].

AEs, defined as grade 3 or higher, were compared between the With-ICI and Without-ICI groups. The meta-analysis for AEs, which were assessed in four [9,10,11,12,13] studies, demonstrated an increased trend in the With-ICI group compared with the Without-ICI group, but the difference did not reach statistical significance, with an OR of 1.56 (95% CI: 1.00–2.43, *p* = 0.05) (Figure 5), and no significant between-study heterogeneity was observed (χ^2^ = 6.57, I^2^ = 54%, *p* = 0.09). A significant publication bias was detected by visual inspection of the funnel plot (Supplementary Figure S1D) and Egger’s test (*p* = 0.891).

## 4. Discussion

This systematic review and meta-analysis of four [9,10,11,12,13] studies yielding a relatively large sample size (*n* = 986) evaluated the additional efficacy of ICIs on cytotoxic chemotherapy plus bevacizumab in patients with mCRC regardless of MMR status. To the best of our knowledge, this is the first meta-analysis to investigate this unmet medical need. This meta-analysis demonstrated that the addition of ICIs significantly improved PFS. Subgroup analyses revealed that this benefit was particularly evident in the first-line treatment setting and among patients treated with anti-PD-L1 antibodies, suggesting that treatment context and ICI class may influence clinical outcomes.

Immunotherapy has enhanced survival in mCRC patients with dMMR tumors (approximately 5% of cases) [2]. However, efforts to replicate these benefits in pMMR tumors have met with limited success, and no RCT to date has confirmed the efficacy of ICIs alone [3,21]. pMMR CRC has low tumor mutation burden (TMB) and reduced neoantigen generation, leading to poor tumor-infiltrating lymphocyte infiltration compared with dMMR CRC [22]. In pMMR CRC, an immunosuppressive tumor microenvironment (TME), rich in tumor-associated macrophages (TAMs), contributes to resistance to ICIs [23]. TAMs, particularly the M2-polarized phenotype, suppress cytotoxic T-cell activity and foster angiogenesis. Notably, preclinical data suggest that VEGF pathway inhibition can reduce M2-TAM prevalence or reprogram them toward a pro-inflammatory M1 phenotype, thereby improving tumor microenvironment immunogenicity and potentially enhancing response to ICIs [24]. Activation of the Wnt/β-catenin pathway, which is mainly due to APC mutations, impairs dendritic cell function and T-cell recruitment. Other mutations, such as RNF43 and R-spondin fusions, further enhance Wnt signaling. These factors create an “immune-cold” environment that limits immunotherapy efficacy [23,25]. Therefore, efforts should be directed to molecular pathways to enhance T-cell recruitment to the TME, thereby converting pMMR CRC into an “immune hot” tumor that may facilitate its response to ICIs.

Dual inhibition of the VEGF and PD-1/PDL-1 axes has yielded clinical activity in various solid tumors, including mCRC [26,27,28]. VEGF inhibition can hinder the expression of immunosuppressive molecules; as a result, they contribute to the restoration of the immunosuppressive TME [29]. Combining ICIs with angiogenesis-targeting tyrosine kinase inhibitors (TKIs) such as regorafenib and fruquintinib could help overcome the immunotherapy resistance seen in pMMR mCRC. A recent meta-analysis published in 2024 demonstrated favorable, but not impactful, effectiveness with a notable safety profile of the combination of ICIs and TKIs compared with TKIs alone [30]. Although the VEGF pathway plays a role in sustaining immunosuppression within the TME, its activity is less prominent in CRC than in renal cell carcinoma [31], where ICIs plus anti-VEGF TKI therapy has become the standard treatment approach. While all the included studies in the present analysis were designed to evaluate the addition of ICIs to a VEGF inhibitor-based regimen, there have also been clinical trials with the reverse design, evaluating the addition of VEGF inhibitors to ICI-based therapy. Notably, in biliary tract cancer, such a trial design also demonstrated positive results in PFS, supporting the potential benefit of this combination approach [32].

As another approach, cytotoxic agents have been combined with ICIs to leverage their direct cytotoxic effects by releasing potential neoantigens into the circulation to trigger the priming of T cells. This approach has been successful in certain solid tumors, including lung [33], gastric [34], and triple-negative breast cancer [35]. However, in most studies, combining 5-FU–based therapy with ICIs failed to achieve comparable success in mCRC, leading to the discontinuation of these trials in later-line settings [12]. Therefore, earlier lines, especially the first line, are attractive for determining the benefits of this approach. In fact, three [9,10,11,13] of the four [9,10,11,12,13] included studies in this meta-analysis were subjected to earlier lines, and only one in the first-line setting (AtezoTRIBE) [9,10] using an intensive triplet regimen demonstrated a significant improvement in PFS individually. In contrast, another (CheckMate 9X8) [11] study in the first-line setting using a standard doublet regimen failed to show a significant PFS improvement. However, no clear evidence exists that more intensive regimens confer superior immunomodulatory effects compared with conventional doublets, even when a PD-1 inhibitor is used instead of a PD-L1 inhibitor. It is noteworthy that the relatively short follow-up period (e.g., 19.9 months) in the AtezoTRIBE study raised concerns regarding the positive results; however, recently published updated data confirmed the consistency of these findings [10].

Building on these insights, combination strategies targeting both tumor vasculature and the immunosuppressive microenvironment have attracted growing interest. VEGF inhibitors like bevacizumab may normalize vasculature and enhance immune cell infiltration, while chemotherapy promotes antigen release via immunogenic cell death [29]. These effects could synergize with ICIs by boosting T-cell activation and overcoming immune evasion in pMMR mCRC. Recent translational studies support this approach, highlighting the potential of multi-modal therapy to improve outcomes in this setting [9,10]. Supplementary Figure S4 illustrates this concept. Further biomarker-driven trials are needed to refine patient selection and validate these strategies clinically.

Although the integrated PFS result of this meta-analysis was preferable in the combination approach, the results of the included studies were not completely inconsistent. Hence, the identification of a precise and well-validated biomarker is required [36,37]. The AtezoTRIBE [9,10] study demonstrated that higher Immunoscore values, which provide a synthetic description of the spatial distribution of the immune contexture, were associated with greater immunotherapy benefits in pMMR CRC. In addition, high TMB was also a candidate, but the small population in pMMR CRC hampered the validation of its predictive efficacy. In the pMMR mCRC, patients with Immunoscore-high and/or TMB-high tumors are identified as a subgroup of interest to further develop this treatment [10].

Although this meta-analysis demonstrated a significant improvement in PFS, no statistically significant benefit was observed for OS. Several factors may explain these results. First, the relatively short follow-up durations in the included trials might have been insufficient to capture meaningful differences in OS. However, a sensitivity analysis excluding the MODUL study [13], which had the shortest follow-up period, could not confirm that the lack of OS benefit was attributable to follow-up duration. Second, the modest sample sizes and clinical heterogeneity among studies, including differences in treatment lines, chemotherapy regimens, and ICI types, could have diluted any potential survival benefit and response. Third, the lack of OS benefit may also reflect the impact of subsequent therapies after disease progression, which vary across trials and could confound OS outcomes. Moreover, the significant publication bias detected for OS suggests that unpublished negative studies may exist, potentially influencing the pooled results.

This meta-analysis has several limitations: (1) Only four RCTs without a phase 3 trial were included, limiting the statistical power of the overall and subgroup analyses. Future meta-analyses incorporating full-scale phase 3 trials will be essential to confirm and refine these preliminary observations. (2) Considerable heterogeneity existed among the studies in terms of patient selection, therapeutic lines (first line vs. later line), cytotoxic agents, and ICI types. Among these, the inconsistency in the treatment lines may have had the greatest impact. Three of the four studies were conducted in the first-line setting [9,10,11,13], and our subgroup analysis demonstrated a statistically significant improvement in PFS in this subgroup (HR, 0.83; 95% CI, 0.70–0.99; *p* = 0.04) (Supplementary Figure S2). (3) Both PD-1 and PD-L1 inhibitors were collectively analyzed as ICIs. Although mechanistic differences exist, for example, PD-L1 inhibitors not only block the PD-1/PD-L1 axis but also promote lysosomal degradation of PD-L1 in macrophages [36,37], a subgroup analysis separating PD-1 and PD-L1 inhibitors demonstrated that the PD-L1 subgroup (comprising three of the four studies) showed a significant PFS benefit (HR = 0.82, 95% CI: 0.69–0.97, *p* = 0.02), whereas the PD-1 subgroup (one study) did not reach statistical significance (HR = 0.81, 95% CI: 0.53–1.23, *p* = 0.33). However, no statistically significant subgroup difference was observed (*p* for interaction = 0.96), indicating consistency across ICI classes (Supplementary Figure S3). (4) All included studies enrolled both pMMR and a small number of dMMR tumors, as the efficacy of ICIs in dMMR CRC had not been fully established when these studies were initiated. As a result, the inclusion of dMMR cases may have contributed to the overestimation of treatment effects and should be interpreted with caution. However, the MMR status was balanced between treatment arms, and consistent results were observed in pMMR subgroup analyses [9,10,12,13]. (5) Although the statistical heterogeneity across outcomes was low (I^2^ = 0–54%), substantial clinical heterogeneity was present, including differences in chemotherapy backbones, ICI classes, and therapeutic lines. This clinical variability may limit generalizability. Additionally, publication bias was suggested for OS, as shown by funnel plot asymmetry and Egger’s test. These findings should be interpreted with caution, and the potential overestimation of OS should be acknowledged. (6) Due to the small number of available trials, publication bias could not be entirely excluded.

## 5. Conclusions

In conclusion, this meta-analysis suggested that the combination of an ICI with cytotoxic chemotherapy and bevacizumab would demonstrate a potential progression-free survival benefit and an acceptable safety profile in the management of patients with pMMR mCRC. However, three [11,12,13] of the four [9,10,11,12,13] included studies did not show a statistically significant improvement in PFS, and this meta-analysis did not demonstrate a significant benefit in OS. Therefore, further research is needed to accumulate more robust evidence and to define optimal strategies and combinations for integrating ICIs into the treatment plans of patients with pMMR mCRC.

## Figures and Tables

**Table 1:** Characteristics of included studies in the meta-analysis.

Author	Year	Country	Study Design	Study Period	Institutions	Total Cases (With/Without ICI)	dMMR or MSI-H (With/Without ICI)	Treatment Line	ICI	Backbone Chemotherapy	Primary Endpoint	Median Follow Up (With/Without ICI)
AtezoTRIBE (Antoniotti et al.) [9,10]	2022, 2024	Italy	RCT (Phase 2, Open-label)	2018–2020	Multi	218 (145/73)	(7%/6%)	First-line	Atezolizumab	FOLFOXIRI + Bevacizumab	PFS	19.9 M (2022) 45.2 M (2024)
BACCI (Mettu et al.) [12]	2022	United States	RCT (Phase 2, double-blind)	2017–2018	Multi	128 (82/46)	7.3% (7.7%/6.7%)	Second-line or later	Atezolizumab	Capecitabine + Bevacizumab	PFS	20.9 M (for PFS)
MODUL (Tabernero et al.) [13]	2022	Europe, Asia, Africa, America	RCT (Phase 2, Open-label)	2015–2016	Multi	445 (297/148)	(2.0%/1.6%)	First-line	Atezolizumab	Fluoropyrimidine + Bevacizumab	PFS	10.5 M (10.6 M/10.4 M)
CheckMate 9X8 (Lenz et al.) [11]	2024	United States	RCT (Phase 2/3, Open-label)	2018–2019	Multi	195 (127/68)	(5%/10%)	First-line	Nivolumab	mFOLFOX6 + Bevacizumab	PFS	21.5 M (minimum) (23.7 M/23.2 M)

ICI: immune-check point inhibitor, MMR: mismatch repair, MSI: microsatellite instability, RCT: randomized controlled trial, Multi: multi-institution, PFS: progression-free survival, and M: month.

**Table 2:** Risk of bias assessment of included studies in the meta-analysis.

Author	Randomization	Allocation Concealment	Blinding	Missing Data	Selective Reporting	Overall Risk
AtezoTRIBE (Antoniotti et al.) [9,10]	Low	Low	Some concerns	Low	Low	Some concerns
BACCI (Mettu et al.) [12]	Low	Low	Low	Low	Low	Low
MODUL (Tabernero et al.) [13]	Low	Low	Some concerns	Low	Low	Some concerns
CheckMate 9X8 (Lenz et al.) [11]	Low	Low	Some concerns	Low	Low	Some concerns

**Table 3:** Level of evidence according to the GRADE criteria.

Outcome	Overall Quality	Risk of Bias	Inconsistency	Indirectness	Imprecision	Publication Bias	Comments
PFS	Low	⚫⚫⚫⚪ (Some concerns)	⚫⚫⚫⚫ (No concerns)	⚫⚫⚫⚫ (No concerns)	⚫⚫⚪⚪ (Serious)	⚫⚫⚫⚫ (No concerns)	Wider confidence interval and lack of blinding in some trials reduce certainty.
OS	Low	⚫⚫⚫⚪ (Some concerns)	⚫⚫⚫⚫ (No concerns)	⚫⚫⚫⚫ (No concerns)	⚫⚫⚪⚪ (Serious)	⚫⚫⚪⚪ (Serious)	No significant difference in mortality observed; wide Confidence interval reduces certainty.
ORR	Low	⚫⚫⚫⚪ (Some concerns)	⚫⚫⚫⚫ (No concerns)	⚫⚫⚫⚫ (No concerns)	⚫⚫⚪⚪ (Serious)	⚫⚫⚫⚫ (No concerns)	Improvement in response rate observed, but inconsistency across trials.

PFS: progression-free survival, OS: overall survival, ORR: overall response rate.

## Data Availability

The original contributions presented in this study are included in the article/Supplementary Materials. Further inquiries can be directed to the corresponding author.

## Supplementary Materials

The following supporting information can be downloaded at https://www.mdpi.com/article/10.3390/cancers17152538/s1; Supplementary Figure S1. Funnel plots assessing publication bias for (A) progression-free survival, (B) overall survival, (C) overall response rate, and (D) serious adverse events. Supplementary Figure S2. Forest plot of progression-free survival (PFS) by treatment line (first line vs. subsequent line). Supplementary Figure S3. Forest plot of progression-free survival (PFS) by immune checkpoint inhibitor (ICI) class (PD-1 vs. PD-L1 inhibitors). Supplementary Figure S4. Schematic model of the synergistic effect of enhanced immune infiltration, antigen release, and T-cell activation overcoming immune resistance in pMMR mCRC.

## References

[1] Trullas A., Delgado J., Genazzani A., Mueller-Berghaus J., Migali C., Muller-Egert S., Zander H., Enzmann H., Pignatti F. (2021). The EMA assessment of pembrolizumab as monotherapy for the first-line treatment of adult patients with metastatic microsatellite instability-high or mismatch repair deficient colorectal cancer. ESMO Open.

[2] Andre T., Shiu K.K., Kim T.W., Jensen B.V., Jensen L.H., Punt C., Smith D., Garcia-Carbonero R., Benavides M., Gibbs P. (2020). Pembrolizumab in Microsatellite-Instability-High Advanced Colorectal Cancer. N. Engl. J. Med..

[3] Le D.T., Uram J.N., Wang H., Bartlett B.R., Kemberling H., Eyring A.D., Skora A.D., Luber B.S., Azad N.S., Laheru D. (2015). PD-1 Blockade in Tumors with Mismatch-Repair Deficiency. N. Engl. J. Med..

[4] Finn R.S., Qin S., Ikeda M., Galle P.R., Ducreux M., Kim T.Y., Kudo M., Breder V., Merle P., Kaseb A.O. (2020). Atezolizumab plus Bevacizumab in Unresectable Hepatocellular Carcinoma. N. Engl. J. Med..

[5] Rini B.I., Powles T., Atkins M.B., Escudier B., McDermott D.F., Suarez C., Bracarda S., Stadler W.M., Donskov F., Lee J.L. (2019). Atezolizumab plus bevacizumab versus sunitinib in patients with previously untreated metastatic renal cell carcinoma (IMmotion151): A multicentre, open-label, phase 3, randomised controlled trial. Lancet.

[6] Socinski M.A., Jotte R.M., Cappuzzo F., Orlandi F., Stroyakovskiy D., Nogami N., Rodriguez-Abreu D., Moro-Sibilot D., Thomas C.A., Barlesi F. (2018). Atezolizumab for First-Line Treatment of Metastatic Nonsquamous NSCLC. N. Engl. J. Med..

[7] Hegde P.S., Wallin J.J., Mancao C. (2018). Predictive markers of anti-VEGF and emerging role of angiogenesis inhibitors as immunotherapeutics. Semin. Cancer Biol..

[8] Wallin J.J., Bendell J.C., Funke R., Sznol M., Korski K., Jones S., Hernandez G., Mier J., He X., Hodi F.S. (2016). Atezolizumab in combination with bevacizumab enhances antigen-specific T-cell migration in metastatic renal cell carcinoma. Nat. Commun..

[9] Antoniotti C., Rossini D., Pietrantonio F., Catteau A., Salvatore L., Lonardi S., Boquet I., Tamberi S., Marmorino F., Moretto R. (2022). Upfront FOLFOXIRI plus bevacizumab with or without atezolizumab in the treatment of patients with metastatic colorectal cancer (AtezoTRIBE): A multicentre, open-label, randomised, controlled, phase 2 trial. Lancet Oncol..

[10] Antoniotti C., Rossini D., Pietrantonio F., Salvatore L., Lonardi S., Tamberi S., Marmorino F., Moretto R., Prisciandaro M., Tamburini E. (2024). Upfront Fluorouracil, Leucovorin, Oxaliplatin, and Irinotecan Plus Bevacizumab With or Without Atezolizumab for Patients with Metastatic Colorectal Cancer: Updated and Overall Survival Results of the ATEZOTRIBE Study. J. Clin. Oncol..

[11] Lenz H.J., Parikh A., Spigel D.R., Cohn A.L., Yoshino T., Kochenderfer M., Elez E., Shao S.H., Deming D., Holdridge R. (2024). Modified FOLFOX6 plus bevacizumab with and without nivolumab for first-line treatment of metastatic colorectal cancer: Phase 2 results from the CheckMate 9X8 randomized clinical trial. J. Immunother. Cancer.

[12] Mettu N.B., Ou F.S., Zemla T.J., Halfdanarson T.R., Lenz H.J., Breakstone R.A., Boland P.M., Crysler O.V., Wu C., Nixon A.B. (2022). Assessment of Capecitabine and Bevacizumab with or Without Atezolizumab for the Treatment of Refractory Metastatic Colorectal Cancer: A Randomized Clinical Trial. JAMA Netw. Open.

[13] Tabernero J., Grothey A., Arnold D., de Gramont A., Ducreux M., O’Dwyer P., Tahiri A., Gilberg F., Irahara N., Schmoll H.J. (2022). MODUL cohort 2: An adaptable, randomized, signal-seeking trial of fluoropyrimidine plus bevacizumab with or without atezolizumab maintenance therapy for BRAF(wt) metastatic colorectal cancer. ESMO Open.

[14] Page M.J., McKenzie J.E., Bossuyt P.M., Boutron I., Hoffmann T.C., Mulrow C.D., Shamseer L., Tetzlaff J.M., Akl E.A., Brennan S.E. (2021). The PRISMA 2020 statement: An updated guideline for reporting systematic reviews. BMJ.

[15] Otsuka T., Matsuyama K. (2024). Nippon Medical School’s Ethical Review Processes for Studies Involving Human Subjects. J. Nippon Med. Sch..

[16] Balshem H., Helfand M., Schunemann H.J., Oxman A.D., Kunz R., Brozek J., Vist G.E., Falck-Ytter Y., Meerpohl J., Norris S. (2011). GRADE guidelines: 3. Rating the quality of evidence. J. Clin. Epidemiol..

[17] Parmar M.K., Torri V., Stewart L. (1998). Extracting summary statistics to perform meta-analyses of the published literature for survival endpoints. Stat. Med..

[18] DerSimonian R., Laird N. (1986). Meta-analysis in clinical trials. Control. Clin. Trials.

[19] Higgins J.P., Thompson S.G., Deeks J.J., Altman D.G. (2003). Measuring inconsistency in meta-analyses. BMJ.

[20] Egger M., Davey Smith G., Schneider M., Minder C. (1997). Bias in meta-analysis detected by a simple, graphical test. BMJ.

[21] Chen E.X., Jonker D.J., Loree J.M., Kennecke H.F., Berry S.R., Couture F., Ahmad C.E., Goffin J.R., Kavan P., Harb M. (2020). Effect of Combined Immune Checkpoint Inhibition vs Best Supportive Care Alone in Patients with Advanced Colorectal Cancer: The Canadian Cancer Trials Group CO.26 Study. JAMA Oncol..

[22] Guler I., Askan G., Klostergaard J., Sahin I.H. (2019). Precision medicine for metastatic colorectal cancer: An evolving era. Expert Rev. Gastroenterol. Hepatol..

[23] Sahin I.H., Akce M., Alese O., Shaib W., Lesinski G.B., El-Rayes B., Wu C. (2019). Immune checkpoint inhibitors for the treatment of MSI-H/MMR-D colorectal cancer and a perspective on resistance mechanisms. Br. J. Cancer..

[24] Wang S., Wang J., Chen Z., Luo J., Guo W., Sun L., Lin L. (2024). Targeting M2-like tumor-associated macrophages is a potential therapeutic approach to overcome antitumor drug resistance. npj Precis. Oncol..

[25] Zhou Y., Xu J., Luo H., Meng X., Chen M., Zhu D. (2022). Wnt signaling pathway in cancer immunotherapy. Cancer Lett..

[26] Colombo N., Dubot C., Lorusso D., Caceres M.V., Hasegawa K., Shapira-Frommer R., Tewari K.S., Salman P., Hoyos Usta E., Yanez E. (2021). Pembrolizumab for Persistent, Recurrent, or Metastatic Cervical Cancer. N. Engl. J. Med..

[27] Fukuoka S., Hara H., Takahashi N., Kojima T., Kawazoe A., Asayama M., Yoshii T., Kotani D., Tamura H., Mikamoto Y. (2020). Regorafenib Plus Nivolumab in Patients with Advanced Gastric or Colorectal Cancer: An Open-Label, Dose-Escalation, and Dose-Expansion Phase Ib Trial (REGONIVO, EPOC1603). J. Clin. Oncol..

[28] Powles T., Plimack E.R., Soulieres D., Waddell T., Stus V., Gafanov R., Nosov D., Pouliot F., Melichar B., Vynnychenko I. (2020). Pembrolizumab plus axitinib versus sunitinib monotherapy as first-line treatment of advanced renal cell carcinoma (KEYNOTE-426): Extended follow-up from a randomised, open-label, phase 3 trial. Lancet Oncol..

[29] Zhao S., Ren S., Jiang T., Zhu B., Li X., Zhao C., Jia Y., Shi J., Zhang L., Liu X. (2019). Low-Dose Apatinib Optimizes Tumor Microenvironment and Potentiates Antitumor Effect of PD-1/PD-L1 Blockade in Lung Cancer. Cancer Immunol. Res..

[30] Li J., Zhu J.X., Zhang Y.X., Li S.Q. (2024). Effectiveness of immune checkpoint inhibitors in combination with tyrosine kinase inhibitors in patients with advanced or metastatic colorectal carcinoma with either mismatch repair proficient or metastatic microsatellite stable disease: A systematic review and meta-analysis. Oncol. Lett..

[31] Zhang W., Vallboehmer D., Mizutomo A. (2006). Differential gene expression levels of vascular endothelial growth factor (VEGF) and its receptors in renal cell cancer and colorectal cancer patients. Cancer Res..

[32] Macarulla T., Ren Z., Chon H.J., Park J.O., Kim J.W., Pressiani T., Li D., Zhukova L., Zhu A.X., Chen M.H. (2025). Atezolizumab Plus Chemotherapy with or Without Bevacizumab in Advanced Biliary Tract Cancer: Clinical and Biomarker Data From the Randomized Phase II IMbrave151 Trial. J. Clin. Oncol..

[33] Novello S., Kowalski D.M., Luft A., Gumus M., Vicente D., Mazieres J., Rodriguez-Cid J., Tafreshi A., Cheng Y., Lee K.H. (2023). Pembrolizumab Plus Chemotherapy in Squamous Non-Small-Cell Lung Cancer: 5-Year Update of the Phase III KEYNOTE-407 Study. J. Clin. Oncol..

[34] Janjigian Y.Y., Shitara K., Moehler M., Garrido M., Salman P., Shen L., Wyrwicz L., Yamaguchi K., Skoczylas T., Campos Bragagnoli A. (2021). First-line nivolumab plus chemotherapy versus chemotherapy alone for advanced gastric, gastro-oesophageal junction, and oesophageal adenocarcinoma (CheckMate 649): A randomised, open-label, phase 3 trial. Lancet.

[35] Cortes J., Rugo H.S., Cescon D.W., Im S.A., Yusof M.M., Gallardo C., Lipatov O., Barrios C.H., Perez-Garcia J., Iwata H. (2022). Pembrolizumab plus Chemotherapy in Advanced Triple-Negative Breast Cancer. N. Engl. J. Med..

[36] Ando F., Kashiwada T., Kuroda S., Fujii T., Takano R., Miyabe Y., Kunugi S., Sakatani T., Miyanaga A., Asatsuma-Okumura T. (2024). Combination of plasma MMPs and PD-1-binding soluble PD-L1 predicts recurrence in gastric cancer and the efficacy of immune checkpoint inhibitors in non-small cell lung cancer. Front. Pharmacol..

[37] Kashiwada T., Takano R., Ando F., Kuroda S., Miyabe Y., Owada R., Miyanaga A., Asatsuma-Okumura T., Hashiguchi M., Kanazawa Y. (2024). Lysosomal degradation of PD-L1 is associated with immune-related adverse events during anti-PD-L1 immunotherapy in NSCLC patients. Front. Pharmacol..

