# Supplementary material for: Efficacy of Adding Immune Checkpoint Inhibitors to Chemotherapy Plus Bevacizumab in Metastatic Colorectal Cancer: A Meta-Analysis of Randomized Controlled Trials
Source: Cancers (Basel). 2025 Jul 31;17(15):2538. doi: 10.3390/cancers17152538 (PMC12346875; doi:10.3390/cancers17152538)
Supplement: Supplementary file 1 [file cancers-17-02538-s001.zip › cancers-3739537-supplementary.pdf]

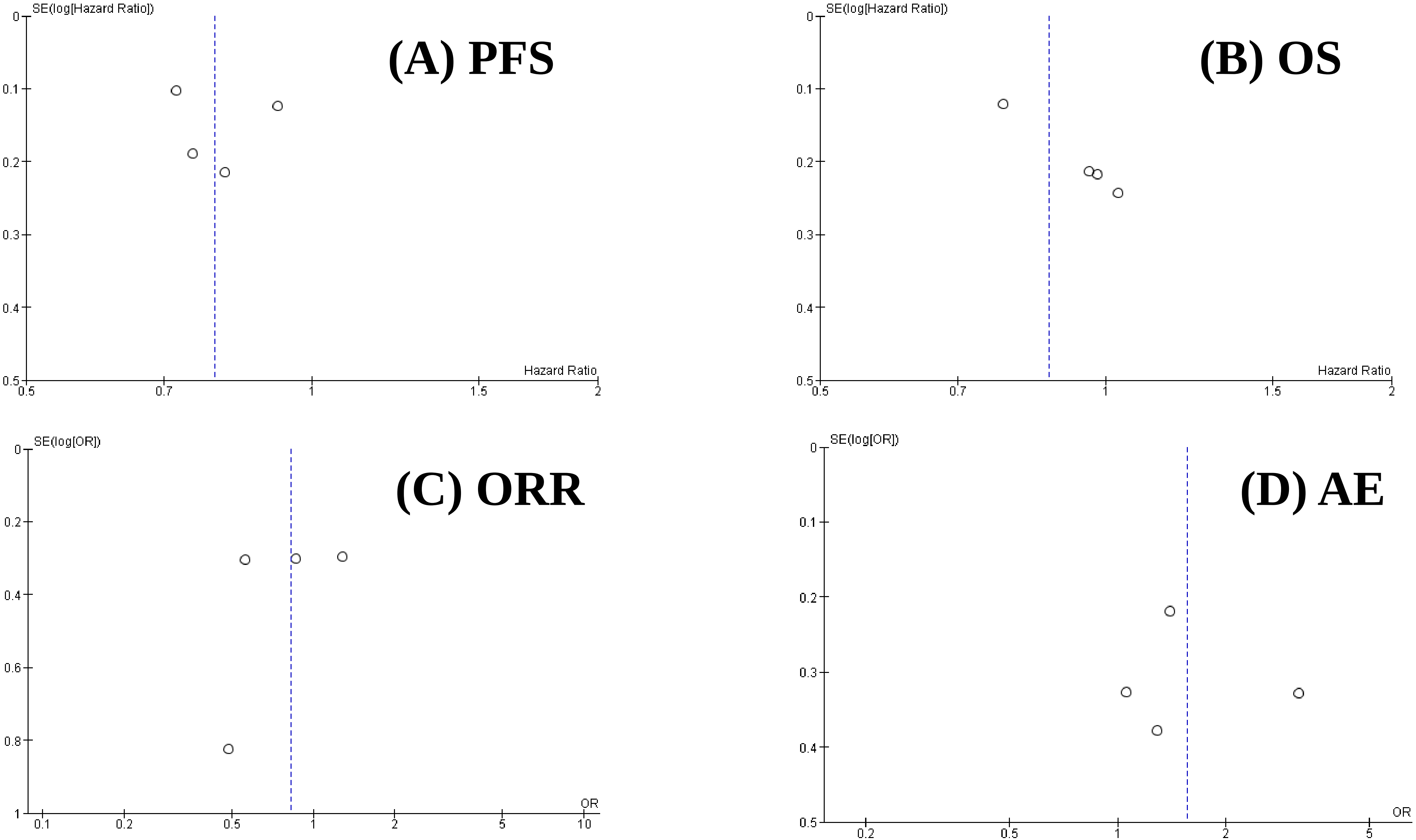

Supplementary Figure S1

Study or Subgroup	log[Hazard Ratio]	SE	Weight	Hazard Ratio IV, Random, 95% CI
**1.12.1 First line setting**				
AtezoTRIBE (Antoniotti et al., 2022)	-0.3285	0.1562	26.3%	0.72 [0.53, 0.98]
CheckMate 9X8 (Lenz et al., 2024)	-0.211	0.215	13.9%	0.81 [0.53, 1.23]
MODUL (Tabernero et al.,2022)	-0.0833	0.1238	41.8%	0.92 [0.72, 1.17]
**Subtotal (95% CI)**			**82.0%**	**0.83 [0.70, 0.99]**
Heterogeneity: Tau² = 0.00; Chi² = 1.53, df = 2 (P = 0.46); I² = 0%				
Test for overall effect: Z = 2.07 (P = 0.04)				
**1.12.2 Subsequent line setting**				
BACCI (Mettu et al., 2022)	-0.2876	0.1888	18.0%	0.75 [0.52, 1.09]
**Subtotal (95% CI)**			**18.0%**	**0.75 [0.52, 1.09]**
Heterogeneity: Not applicable				
Test for overall effect: Z = 1.52 (P = 0.13)				
**Total (95% CI)**			**100.0%**	**0.82 [0.70, 0.96]**
Heterogeneity: Tau² = 0.00; Chi² = 1.78, df = 3 (P = 0.62); I² = 0%				
Test for overall effect: Z = 2.53 (P = 0.01)				
Test for subgroup differences: Chi² = 0.25, df = 1 (P = 0.62), I² = 0%				

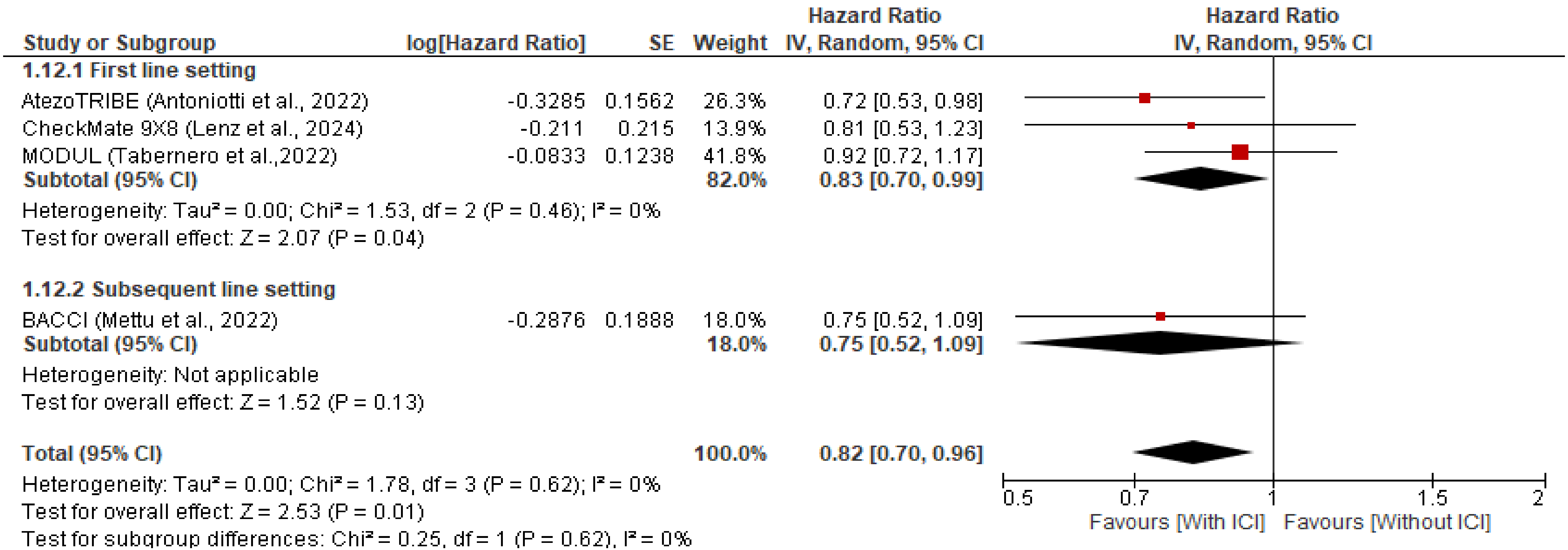

Supplementary Figure S2

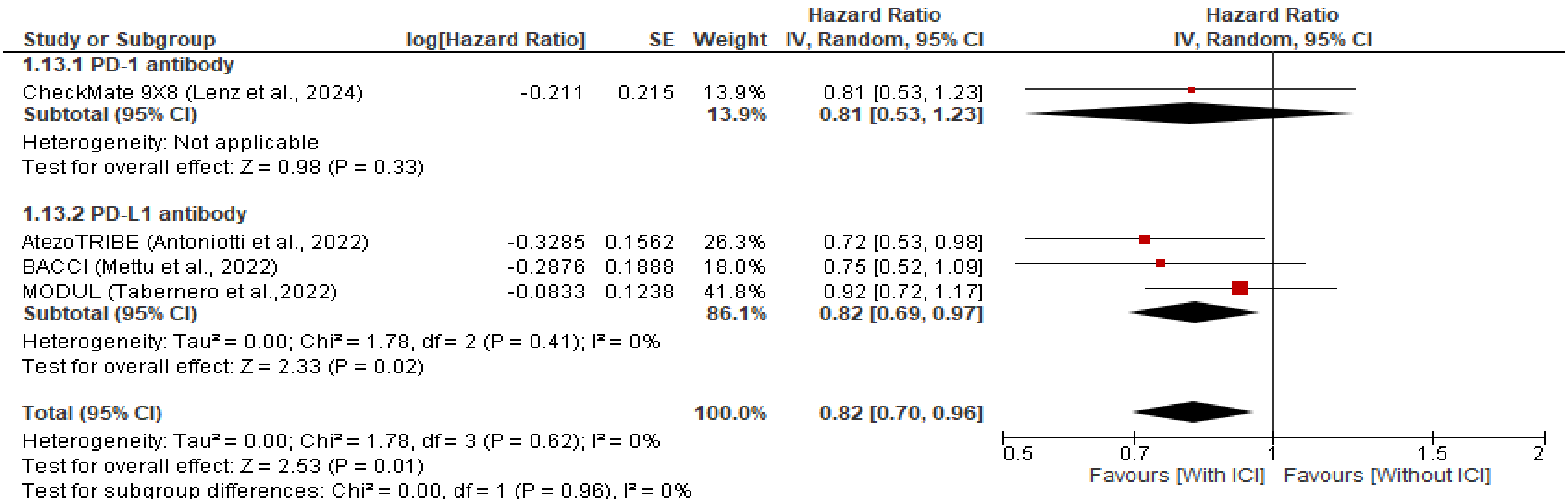

Supplementary Figure S3

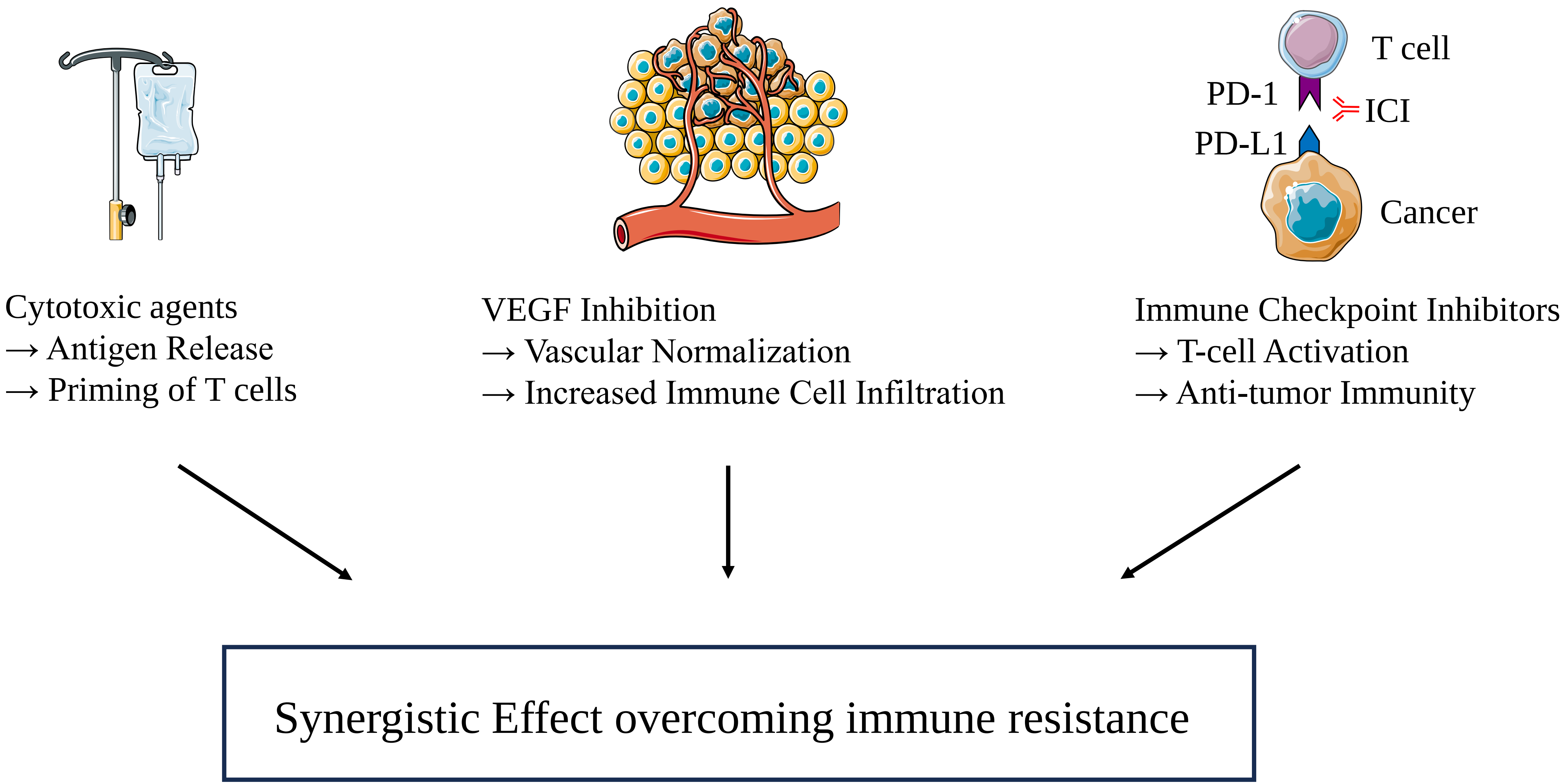

Supplementary Figure S4
